# Analysis of proliferative activity in oral gingival epithelium in immunosuppressive medication induced gingival overgrowth

**DOI:** 10.1186/1746-160X-2-13

**Published:** 2006-05-19

**Authors:** Şule Bulut, Hilal Uslu, B Handan Özdemir, Ömer Engin Bulut

**Affiliations:** 1 Department of Periodontology, University of Baskent, Faculty of Dentistry, Ankara, Türkiye; 2 Department of Periodontology, University of Baskent, Faculty of Dentistry, Ankara, Türkiye; 3 Department of Pathology, University of Baskent, Faculty of Medicine, Ankara, Türkiye; 4 Department of Oral and Maxillofacial Surgery, University of Baskent, Faculty of Dentistry, Ankara, Türkiye

## Abstract

**Background:**

Drug-induced gingival overgrowth is a frequent adverse effect associated principally with administration of the immunosuppressive drug cyclosporin A and also certain antiepileptic and antihypertensive drugs. It is characterized by a marked increase in the thickness of the epithelial layer and accumulation of excessive amounts of connective tissue. The mechanism by which the drugs cause gingival overgrowth is not yet understood. The purpose of this study was to compare proliferative activity of normal human gingiva and in cyclosporine A-induced gingival overgrowth.

**Methods:**

Gingival samples were collected from 12 generally healthy individuals and 22 Cyclosporin A-medicated renal transplant recipients. Expression of proliferating cell nuclear antigen was evaluated in formalin-fixed, paraffin-embedded gingival samples using an immunoperoxidase technique and a monoclonal antibody for this antigen.

**Results:**

There were differences between the Cyclosporin A group and control group in regard to proliferating cell nuclear antigen and epithelial thickness. In addition, the degree of stromal inflammation was higher in the Cyclosporin A group when compared with the control group.

**Conclusion:**

The results suggest that the increased epithelial thickness observed in Cyclosporin A-induced gingival overgrowth is associated with increased proliferative activity in keratinocytes.

## Background

Drug-induced gingival overgrowth (DGO) is an adverse effect of certain medicines such a immunosuppressive agents, antiepileptics, and calcium (Ca 2+) channel blockers [1]. However, the exact mechanisms underlying the pathogenesis of drug-induced enlargement remain unclear, particularly of the immunosuppressive agents. In general, DGO is clinically associated with gingival inflammation produced by microbial plaque. This unwanted side effect may greatly influence the clinical course of gingival tissues and subsequent systemic health, if complicated. For this reason, it is obvious that a better understanding of the pathogenesis of DGO is one of the important subjects in clinical periodontology.

The gingival epithelium plays an important role in protecting against both bacterial infection and mechanical trauma [2]. Keratinocytes are the dominant cells of the epidermis, constituting 90% of the gingival cell population [3]. The self-renewal capacity of the gingival epithelium contributes to gingival defense, since continuous desquamation of superficial epithelial cells prevents bacterial colonization. Therefore, changes in the turnover rate of gingival epithelium may affect progression of periodontal disease [4-6].

Histologically, drug-induced gingival overgrowth is associated with thickening of epithelium with elongated rete pegs and fibrosis in the lamina propria, with increased numbers of fibroblasts. [7] Ramon et al. [8] showed that the thickness of the oral epithelium in nifedipine-medicated patients was some 5 to10 times greater than in healthy controls. The volume density of oral epithelium is significantly increased in CsA-induced gingival overgrowth as compared with non-medicated controls [9]. The epithelial thickening induced by nifedipine [7] and CsA is related to thickening of the spinous layer [9]. Results of clinical studies indicate that gingival inflammation increases the incidence and severity of gingival overgrowth in nifedipine-and/or CsA-mediated patients [1]. Odile et. al [10] showed that keratinocytes cultured from clinically healthy and inflamed human gngival tissue explants proliferate at different rates. The mean proliferation rate in the minimally to slightly inflamed group was significantly higher than in the moderately and severely inflamed groups. Nurmenniemi etal. [12] found that the increased epithelial thickness observed in nifedipine- and cyclosporin A-induced gingival overgrowth is associated with increased mitotic activity, especially in oral epithelium.

Proliferating cell nuclear antigen (PCNA) is a 36-kDa acidic nonhistone nuclear protein that bears an important function in DNA synthesis [12,13]. Its cell concentration is directly correlated with the proliferative state of the cell, increasing through G1, peaking at the G1/S phase interface, decreasing through G2, and reaching low levels in M-phase and interphase [13-15]. PCNA expression, therefore, is believed to be a good indicator of cell proliferation. Casasco etal. [15] have suggested that PCNA antibodies may be useful tools for studying cell kinetics in human oral tissues in normal as well as in pathological situations.

All these data indicate that the association between CsA-induced gingival overgrowth and proliferative activity seem to a relevant role in the pathogenesis of DOG. The aim of this study was to compare the proliferative activity of keratinocytes in plaque induced gingivitis and in cyclosporine A-induced gingival overgrowth (CsA-induced GO).

## Methods

### Patient selection and collection of gingival tissues

Gingival biopsies (one per person) were harvested from 22 renal transplant recipients (8 men and 14 women; mean age, 36.4 ± 13.3 years) diagnosed with CsA-induced gingival enlargement and from 12 systemically healthy subjects (7 males and 5 females; mean age 27.0 ± 16.0 years) with plaque-induced gingivitis. All kidney recipients had severe gingivitis but no signs of periodontitis. Samples of overgrown gingiva from this group were obtained during gingivectomy procedures, all of which all met the guidelines of the Başkent University Ethics Committee. Tissue biopsies from the controls were obtained during routine dental treatment (tooth extraction and gingivoplasty).

All kidney recipients had been taking CsA (200 mg/day), prednisolone (20 mg/day), and doxazosin mesylate (4 mg/day) for approximately 2 years and were still on this regimen. Patients using other drugs known to induce GO were excluded. The healthy individuals with gingivitis had no history of treatment with agents known to cause drug-induced GO. They had not taken antiinflammatory agents, antibiotics, or contraceptives in the previous 6 months. None of the female subjects were pregnant.

Prior to periodontal intervention, each of the 32 study subjects was clinically assessed, and probing depth (PD), gingival index (GI), [16] and plaque index (PI), [17] were recorded.

### Tissue processing and immunohistochemistry

All biopsies were fixed in formalin and embedded in paraffin, and 4-μm – thick sections were cut and stained with hematoxylin-eosin (H&E). All H&E-stained biopsies were evaluated for the presence of hyperplasia of the epithelium and the presence of inflammatory cells in the stroma. The degree of inflammatory cell infiltration in the stroma was graded in 3 groups as follows: grade 1 = inflammatory cells comprising less than 20% of the stroma; grade 2 = inflammatory cells comprising 21%-50% of the stroma; and grade 3 = inflammatory cells comprising more than 51% of the stroma. Four sites were measured within each sample and then mean epithelial thickness was calculated. An ocular grid was used to measure the entire thickness of the epithelium in each case. The epithelial thickness of gingival specimens in two groups was measured as the distance between the granular layer and basal layer of epithelium.

For immunohistochemistry, briefly, 4-μm – thick sections were deparaffinized and mounted on poly-L-lysine-coated slides. Sections in a citrate buffer (0.01 mol/L, pH 6) were heated in a microwave oven for 15 minutes at maximum power (700 W) and then cooled at room temperature for 20 minutes. A standard 3-step immunoperoxidase technique was used to detect PCNA (PC-10, Neomarkers, Fremont, Calif, USA).

About 1000 cells were counted in each case to determine the average PCNA labeling index. The field to be counted was chosen under × 40 magnification from the well-labeled area. The PCNA labeling index was expressed as the percentage ratio of total labeled cells to the total number of cells counted.

### Statistical analysis

Differences between the CsA-treated group and the control group with respect to clinical parameters and histopathological findings were analyzed using the Student *t *and chi-square tests. Differences at p < 0.05 were considered to be significant. Correlations between histopathological findings and clinical parameters were tested using analysis of variance (ANOVA).

## Results

The findings for age, sex, and periodontal status in both groups, and for CsA dosages and blood levels of cyclosporine in the CsA group are presented in Table 1. As expected, patients in the CsA group had significantly higher PD, PI, and GI values than did patients in the control group (*P *< 0.05 for all clinical findings).

**Table 1:** Patient characteristics and periodontal parameters of study population.

	**CsA group**	**Control group**
**Men/Women**	8/14	7/5
**Age (years)**	36.4 ± 13.3	27.0 ± 16.0 years
**Blood CsA (ng/mL)**	202.21 ± 57.21	NA
**Plaque Index **	1.93 ± 0.16*	0.99 ± 0.09
**Gingival Index**	1.88 ± 0.10*	1.08 ± 0.078
**Probing Depth (mm)**	4.25 ± 0.22*	2.49 ± 0.16

*Significant difference p < 0.05

**Figure 1:**
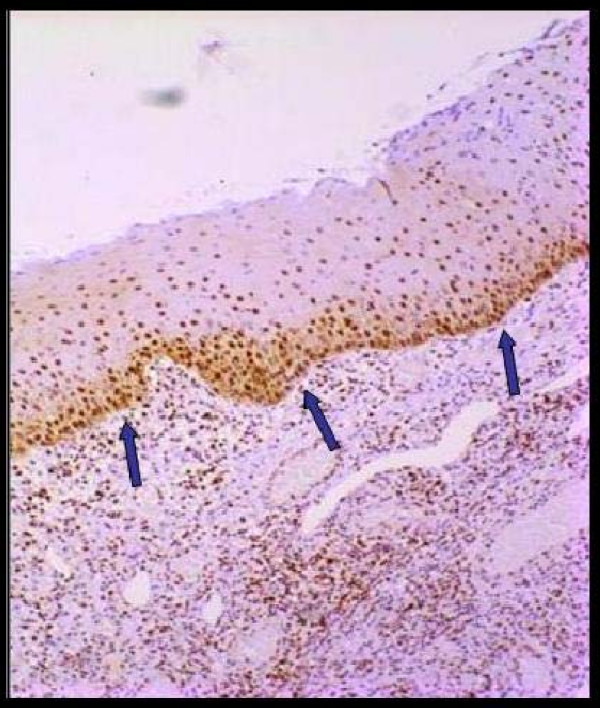
A number of cells showing nuclear staining in the epithelium (control group).

**Figure 2:**
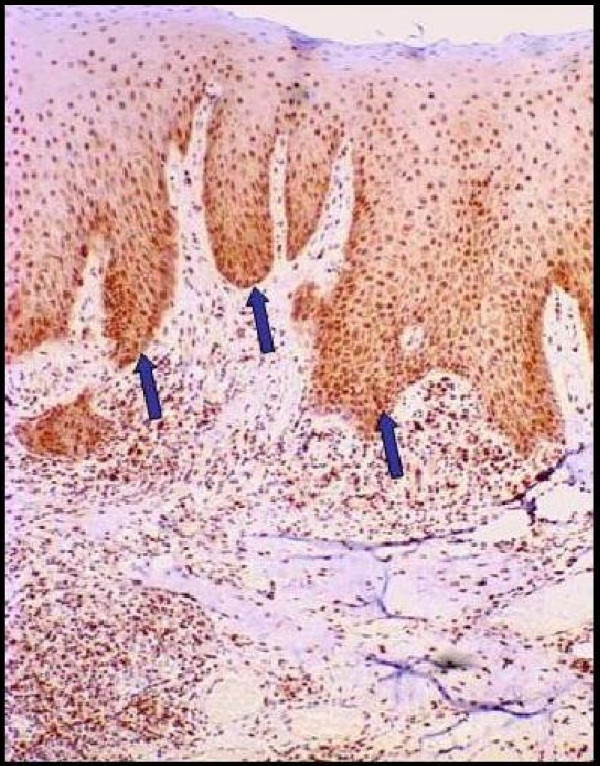
A majority of cells showing nuclear staining with the PCNA antibody (CsA-induced GO group).

**Figure 3:**
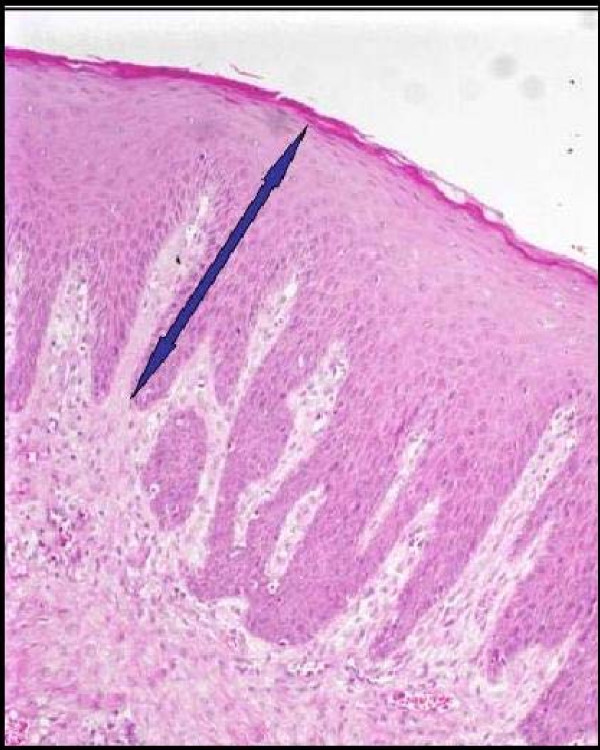
Epithelial thickness in patients in the CsA-induced GO group.

**Figure 4:**
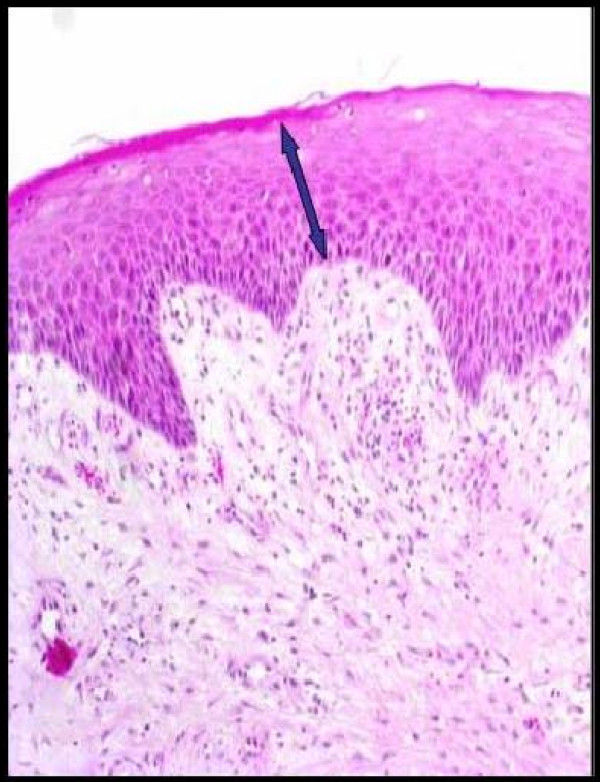
Epithelial thickness in patients in the control group.

The immunohistochemical findings of the two groups are shown in Table 2. There were significant differences between the CsA and control groups with regard to PCNA expression and epithelial thickness (*P *< 0.05 for both) (Figures 1, 2, 3 and 4). In addition, the degree of stromal inflammation was highest in patients in the CsA group when compared with patients in the control group.

**Table 2:** Distribution of immunohistochemical findings in CsA-treated patients and controls

	**CsA Group**	**Control Group**
**PCNA-positive cells**	62.55 ± 3.23 *	30.83 ± 2.80
**Inflammatory cell infiltration grade 1**	18% (n = 4)	83% (n = 10)
**Inflammatory cell infiltration grade 2**	9% (n = 2)	16% (n = 2)
**Inflammatory cell infiltration grade 3**	72% (n = 16)*	-----
**Epithelial thickness (mm)**	0.74 ± 0.03	0.40 ± 0.03

* Significant differences p < 0.05

## Discussion

Several studies have reported CsA-induced GO with regard to certain factors including genetics, duration, dose, serum and salivary concentrations of the drug, oral hygiene, and the age and sex of the patient, with young males being most susceptible [1,18,19]. The mechanisms of GO are unknown. It is, however, possible that gingival tissues may be exposed to higher drug concentrations than other tissues via bloodstream and oral cavity through the crevicular epithelium. CsA appears to influence the growth and function of both the gingival fibroblasts and epithelial cells directly or indirectly. These processes are regulated by cytokines and growth factors, and expression of these mediators and their corresponding receptors is thus likely to be of fundamental importance in the pathophysiology of GO [20-22].

Histologically, drug-induced GO is associated with thickening of the epithelium, as characterized by elongated rete pegs and fibrosis in the lamina propria, and with increased numbers of fibroblasts. Ramon et al [23] have shown that the thickness of the oral epithelium in nifedipine-medicated patients was 5 to 10 times greater than that of healthy controls. The volume density of oral epithelium is significantly increased in CsA-induced gingival overgrowth as compared with nonmedicated controls [8]. In our study, epithelial thickness was higher in CsA-induced GO compared to the control.

Nurmenniemi and coworkers have suggested that epithelial thickening in nifedipine- and CsA-induced GO is associated with mitotic activity in the oral epithelium [11]. In another study, the level of keratinocyted growth factor was elevated in CsA-induced GO, and the authors concluded that keratinocyted growth factor may have an important role in the enhanced epithelial proliferation associated with GO [24]. Our finding that proliferative activity was higher in CsA-induced GO specimens than it was in control specimens agrees with the results of other authors.

In the present study, the CsA-induced GO group displayed singificantly higher values of PI, PD, GI, and epithelial thickness, compared to controls. One previous study demonstrated the presence of increased proliferative activity in oral gingival epithelium during inflammation [25]. Batista de Paula etal. [26] have evaluated the influence of inflammation on immunohistochemical expression of PCNA within the epithelial lining of odontogenic keratocysts. Their results indicated greater proliferative activity in the epithelial cells of inflamed odontogenic keratocysts compared with noninflamed lesions. In view of these findings and those obtained within the conditions of the present study, the increase in epithelial proliferative activity can be regarded as a common response to inflammation.

## Conclusion

Gingival epithelial thickening in CsA-induced GO is associated with increased proliferative activity, and the positive effect of inflammation on epithelial cell proliferation increases with the gingival epithelial thickness. Future studies should clarify the epithelial cell behavior in CsA-induced GO.

## Competing interests

The author(s) declare that they have no competing interests.

## Authors' contributions

SB concieved and coordinated the study andparticipated in the collection of sample and data; and writing the manuscript. HU participated in the collection of samples and writing the manuscript. BHÖ carried out tissue processing and immunohistochemistry. SB, BHÖ and ÖEB analyzed the data. ÖEB participated in the design of the study and performed statistical analysis. All authors read and approved the final manuscript
